# Flow cytometric quantification of tumour endothelial cells; an objective alternative for microvessel density assessment

**DOI:** 10.1038/sj.bjc.6600457

**Published:** 2002-08-01

**Authors:** C I M Baeten, J Wagstaff, I C L Verhoeven, H F P Hillen, A W Griffioen

**Affiliations:** Angiogenesis Laboratory, Research Institute for Growth and Development (GROW), Department of Internal Medicine, University Hospital Maastricht, P.O. Box 5800, 6202 AZ Maastricht, The Netherlands; Department of Pathology, University Hospital Maastricht, P.O. Box 5800, 6202 AZ Maastricht, The Netherlands

**Keywords:** angiogenesis, endothelium, flow cytometry, microvessel density

## Abstract

Assessment of microvessel density by immunohistochemical staining is subject to a considerable inter-observer variation, and this has led to variability in correlation between microvessel density and clinical outcome in different studies. In order to improve the method of microvessel density measurement in tumour biopsies, we have developed a rapid, objective and quantitative method using flow cytometry on frozen tissues. Frozen tissue sections of archival tumour material were enzymatically digested. The single-cell suspension was stained for CD31 and CD34 for flow cytometry. The number of endothelial cells was quantified using light scatter- and fluorescence-characteristics. Tumour endothelial cells were detectable in a single cell suspension, and the percentage of endothelial cells detected in 32 colon carcinomas correlated highly (*r*=0.84, *P*<0.001) with the immunohistochemical assessment of microvessel density. Flow cytometric endothelial cells quantification was found to be more sensitive especially at lower levels of immunohistochemical microvessel density measurement. The current method was found to be applicable for various tumour types and has the major advantage that it provides a retrospective and quantitative approach to the angiogenic potential of tumours.

*British Journal of Cancer* (2002) **87**, 344–347. doi:10.1038/sj.bjc.6600457 www.bjcancer.com

© 2002 Cancer Research UK

## 

Angiogenesis is essential for the outgrowth of tumours and metastasis formation (Griffioen and Molema, 2000). It has been recognised that the angiogenic potential of a tumour contributes to the aggressiveness of that tumour and may therefore be of prognostic importance for various cancers. An increasing number of studies have demonstrated that angiogenesis is predictive for the clinical outcome of the disease, in the sense that an increased level of angiogenesis is inversely related with survival (Weidner *et al*, 1992, 1993). In most of these studies, the angiogenic potential was analysed by immunohistochemical determination of microvessel density (MVD) using antibodies recognising CD31, CD34 or Von Willebrand Factor. This method is currently considered the gold standard for this kind of studies. Although straightforward and routinely performed in most diagnostic laboratories the immunohistochemical MVD measurement has a number of disadvantages. The method is laborious, difficult to quantitate, and suffers from considerable intra- and inter-observer variation. This made an international agreement on performance of MVD-analysis necessary (Vermeulen *et al*, 1996).

It is likely that the difficulties with this method have lead to a substantial number of studies in which no correlation (Brechot *et al*, 1996; Chandrachud *et al*, 1997; Hillen *et al*, 1997) or even an inverse correlation (Sarbia *et al*, 1996; Delahunt *et al*, 1997) was found between angiogenesis and the aggressiveness of the tumour. This urged researchers to quantify MVD by using image-analysis software (Kirchner *et al*, 1996; Wild *et al*, 2000;). Other methods to measure angiogenesis in patients include detection of circulating levels of angiogenic growth factors and soluble adhesion molecules, magnetic resonance imaging and/or positron emission tomography (van Dijke *et al*, 1996; Haubner *et al*, 2001). Major drawbacks of these methods are the costs and the large variation of these measurements in different patients. The current study describes a novel method to efficiently quantify the number of endothelial cells (EC) in frozen tumour biopsies using flowcytometry. The ability to use archival tissue is a major advantage in retrospective fundamental research.

## MATERIALS AND METHODS

### Preparation of single cell suspensions and flow cytometry

Frozen tumour tissues were obtained from the stocks of the Department of Pathology. Of each tumour two 10, 30 and/or 50 μm thick 0.5–1 cm^2^ frozen tissue sections were either directly digested or fixed in 1 ml 1% paraformaldehyde (Sigma, Darmstadt, Germany) in phosphate buffered saline (PBS) or 70% ethanol in water for 1 h at room temperature prior to digestion. After fixation, the sections were washed twice with 4 ml PBS and centrifuged at 400 **g**. The sections were digested by incubation in 1 ml of different concentrations (1, 5 and 10 mg ml^−1^) of collagenase (Life Technologies, Breda, The Netherlands) and dispase (Life Technologies) for time periods of 5–120 min at 37°C during repeated vigorous pipetting. After the digestion, the cells were washed in 10 ml PBS/0.5% bovine serum albumin (BSA), centrifuged at 400 **g** and further processed on ice. Cell preparations were microscopically evaluated for the condition of the single cell suspension. Propidium iodide staining (permeabilisation in 70% ethanol, two washings and resuspending in 20 μg ml^−1^ propidium iodide in PBS) was used to control for intact cells. Cell pellets were incubated with 20 μl of appropriately diluted primary mouse monoclonal antibodies. EC were identified by EN4 anti-human CD31 antibody (1 : 80 hybridoma supernatant, Monosan, Uden, The Netherlands) and QBEND-10 anti-human CD-34 antibody (2 μg ml^−1^, Novocastra, Uden, The Netherlands). After two washings, the cells were incubated with 20 μl biotinylated rabbit anti-mouse Ig antibodies (15 μg ml^−1^, Dako, Uithoorn, The Netherlands) for 1 h. Finally, the cells were stained with phycoerythrin (PE)-conjugated streptavidine (10 μg ml^−1^, Dako) for 30 min. Analysis was performed on a FACS-Calibur (Becton and Dickinson, Mountain View, CA, USA). Both red (585 nm) and green (530 nm) fluorescence, forward light scatter and sidescatter were recorded simultaneously of 10 000 cells. Analysis of data was performed using CellQuest-software (Becton and Dickinson).

### Immunohistochemistry

Five μm serial cryo-sections, adjacent to the sections used for flow cytometry, were put on organosilane coated object slides, air-dried for 24 h at room temperature and fixed with 1% paraformaldehyde (Sigma) for 1 h. Endogenous peroxidase was blocked by incubation in 0,3% H_2_O_2_ in methanol for 30 min. Sections were blocked for non-specific antibody binding with 5% BSA in PBS. The slides were incubated in above mentioned antibodies for 1 h. After incubation with avidine-biotin complex (Vector Laboratories Inc.) for 30 min, the slides were developed with diaminobenzidine (Sigma), counterstained with haematoxylin (Merck, Darmstadt, Germany) and the slides were mounted in entellan (Merck). The microvessel density (MVD) was evaluated as described previously (Hillen *et al*, 1997). In short, two independent observers assessed MVD by either counting of vessels in one high-power field (100×) of three vascular hotspots of a section, or by enumeration of the total amount of blood vessels in three high-power fields randomly selected within a section. Correlation assessment was performed using the Pearson correlation test. Significance of observed differences were assessed using the Student's *t*-test.

## RESULTS

### EC quantification by flow cytometry

We have reported a technique for isolation and phenotyping of endothelial cells (EC) from tumour biopsies (Griffioen *et al*, 1996). For reasons of retrospective research we also wanted to develop this method for frozen tissues. We adapted this technique and the starting point of the investigations was the preparation of thick cryo-sections and subsequent digestion with enzymes. In order to find the optimal thickness of the sections (too thin would lead to damage of too many cells and too thick would lead to inappropriate digestion of the tissue), sections of 10, 30 and 50 μm thickness were prepared. Sections were either directly digested or fixed in 1% paraformaldehyde or 70% ethanol at room temperature prior to digestion. It was found that fixation was needed, since less measurable cells were left after omitting the fixation procedure, due to fragmentation. Fixation with paraformaldehyde gave optimal results (not shown). Sections were digested for different time periods in different concentrations of the collagenase/dispase mix. We found that 30 μm thick sections gave optimal results at all enzyme concentrations. The optimal longevity and concentration of the enzyme treatment was observed to be 15 min with 5 mg ml^−1^ collagenase and 5 mg ml^−1^ dispase (Table 1

**Table 1:**
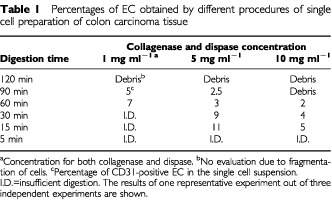
Percentages of EC obtained by different procedures of single cell preparation of colon carcinoma tissue

). Antibodies to CD31 recognised a distinct subpopulation within the single cell suspension (Figure 1A,B

**Figure 1:**
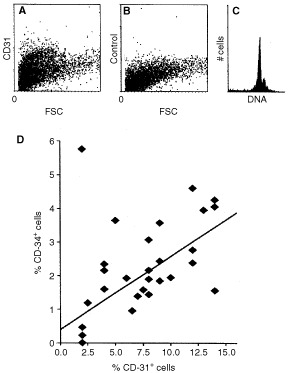
Identification of tumour EC. (**A**) Dot-plot of single cell suspension. Forward scatter (FSC) *vs* indirect CD31 staining (phycoerythrin, vertical axis). (**B**) Conjugate control for the CD31 staining. (**C**) Propidium iodide staining, revealing >95% of cells intact. (**D**) Positive correlation (*r*=0.73, *P*<0.001) of flow-cytometrically identified EC using CD31 and CD34 antibodies in 32 colon carcinomas.

), which contains intact and single cells as determined by propidium iodide staining (Figure 1C). The CD31^+^ cells are of endothelial origin since previous studies, using fresh tissues have demonstrated that these cells are CD105^+^ and ICAM-2^+^ and stain positive after DiI-acetylated-LDL incubation (Griffioen *et al*, 1996). The use of CD34 antibodies resulted in generally lower amounts of stained cells, which was similar to the immunohistochemistry data. However, using flow cytometric analysis a high correlation (*r*=0.73, *P*<0.001) between the percentages of CD31^+^ and CD34^+^ cells was observed (Figure 1D).

This technique of EC quantification in tumour tissues was applicable for tumours of multiple types and origins. Next to colon carcinoma tissue, breast-, lung-, kidney-, head and neck-, ovarian- and testis carcinomas were successfully analysed (not shown).

The analysis of EC percentages in tumour tissues by flow cytometry was found to be highly reproducible. Variation in the percentage of EC obtained by flow cytometry was observed to be less than 5% of the mean, when six independent measurements were performed on one tumour. For example, one tumour analysed using CD31 antibodies revealed a mean percentage of CD31-positive EC of 12.3% (s.d. 0.6%, *n*=6). For MVD assessment by immunohistochemistry a variation of 26% of the mean was observed.

### Flow cytometric quantification of EC correlates with immunohistochemical MVD determination

**Figure 2:**
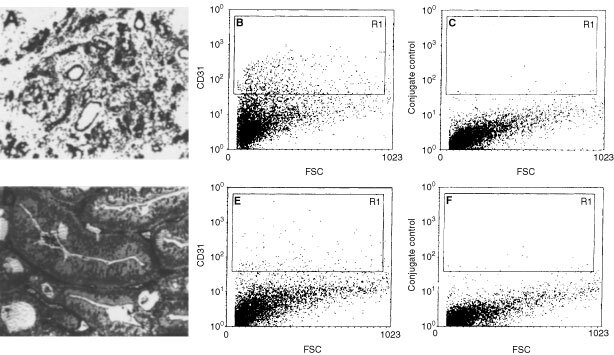
Immunohistochemistry and flow cytometry and of a colon carcinoma with a high (**A**–**C**) and a low (**D**–**F**) vessel density. (**A,D**) Indirect CD31 immunohistochemical staining and counter staining of nuclei with hematoxilin. (**B,E**) Dot-plots of forward scatter *vs* indirect CD31 staining. (**C,F**) conjugate controls.

To investigate whether our method correlates with the immunohistochemical measurement of MVD, a series of 32 cryo-preserved human colon carcinoma tissues (four Dukes A, 15 Dukes B, seven Dukes C and six Dukes D) were analysed. Examples of low vascular density in a well differentiated colon carcinoma and high vascular density in a poorly differentiated colon carcinoma are shown in Figure 2

.

**Figure 3:**
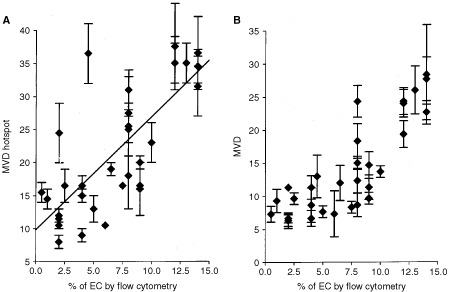
(**A**) Correlation diagram (*r*=0.74, *P*<0.001) of percentage of flow cytometrically identified EC and hotspot MVD measurement (mean number of blood vessels in three hotspots per section). (**B**) Correlation diagram (*r*=0.84, *P*<0.001) of flow cytometrically identified EC and MVD measurement (mean number of blood vessels per high-power field). The mean results of 32 colon carcinomas are shown.

A positive correlation between the percentage of EC and immunohistochemical MVD assessment was observed. A high correlation (*r*=0.74, *P*=0.001) was found when flow cytometric analysis was compared with MVD in vascular hotspots (Figure 3A

). An even higher correlation was found when the overall mean MVD was compared (*r*=0.84, *P*<0.001, Figure 3B). Interestingly, in the latter case, the flow cytometric analysis was found to be more sensitive for tissues with low (<9%) MVD measurements. For tissues with high percentages of EC (>9%), a higher correlation with immunohistochemical MVD assessment was observed (*r*=0.93, *P*<0.002, Figure 3B). A significant correlation with tumour stage was not found. Comparing the different methods, the currently described new flow cytometric technique is most beneficial in the assessment of angiogenesis in tumour tissues.

## DISCUSSION

The capacity of a tumour to induce new blood vessel formation is of key importance for its outgrowth and spread. The golden standard for analysis of angiogenesis, MVD assessment by immunohistochemistry, has a number of disadvantages (see Introduction). The current method is not subject to these problems. It quantifies the number of EC in frozen tissues accurately and objectively. The high correlation with data obtained by immunohistochemical analysis of MVD proved the specificity of our method. Because the current method was set up to determine the number of EC in an entire tissue section, the data obtained should theoretically be most comparable to the data of the immunohistochemical detection of the total mean number of vessels (Figure 3B). Indeed, we found a higher correlation index than with the hotspot countings. In this context two important considerations should be mentioned. Firstly, the size of the blood vessels may be critical. A tissue with a low number of large blood vessels will be scored relatively low by immunohistochemical MVD measurement, whereas, when oxygenation and/or metastasis is concerned, this might be comparable to a higher number of smaller vessels. Enumeration of EC by FACS may level this difference out. Secondly, the nature and composition of the vessel might be critical. Larger vessels may be more mature and consequently contain more extracellular matrix/basal membrane components that can prevent the appropriate digestion and detachment of EC. This may therefore be an intrinsic facilitation of the measurement of angiogenic blood vessels. These two considerations both favour the use of the currently described flow cytometric analysis.

It might be argued that the CD31 antibody also identified other cell types present in the tumour tissues such as leukocytes. However, the immunohistochemistry data did not show staining of leukocytes (see Figure 2). In addition, the leukocyte population has most likely been excluded in the flow cytometric analysis by gating, due to lower scatter characteristics. Therefore, we still favour the use of CD31 antibodies over CD34 antibodies.

Within the group of colon carcinoma tissues, which was selected for representative cases of all Dukes stages, no correlations were found between percentage of EC and Dukes stage, tumour size or survival. This is most likely due to the study group size. Future studies in large numbers of tumour tissues will provide information on clinical parameters, which was beyond the scope of the present study. The current method is potentially also suitable for analysis of tumour EC phenotype. In future studies the number and phenotype of EC in tumour biopsies before and after chemotherapeutic and antiangiogenic treatment modalities will be subject to investigations. This may reveal fundamental insights into the regulation of angiogenesis or alternatively may provide surrogate endpoints of tumour treatments.
